# Association between multi-component initiatives and physical activity-related behaviors: interim findings from the Healthy Schools Healthy Communities initiative

**DOI:** 10.1186/s12889-021-10312-y

**Published:** 2021-02-12

**Authors:** Tamara Vehige Calise, Natalie Spitzer, Laura Ruggiero, Amanda Ryder, Chloe Wingerter, Ashley Hatcher

**Affiliations:** John Snow Inc. (JSI) Healthy Communities, 44 Farnsworth Street, Boston, MA 02210 USA

**Keywords:** Physical activity, Sedentary activity, Youth, Multi-component initiatives, Evaluation

## Abstract

**Background:**

Although successful, assessment of multi-component initiatives (MCIs) prove to be very challenging. Further, rigorous evaluations may not be viable, especially when assessing the impact of MCIs on long-term population-level behavior change (e.g., physical activity (PA) and health outcomes (e.g., childhood obesity). The purpose of this study was to use intensity scoring, to assess whether higher intensity MCIs implemented as part of Healthy Schools Healthy Communities (HSHC) were associated with improved physical activity and reduced sedentary behaviors among youth (dependent variables).

**Methods:**

PA-related interventions were assigned point values based on three characteristics: 1) purpose of initiative; 2) duration; and 3) reach. A MCI intensity score of all strategies was calculated for each school district and its respective community. Multivariate longitudinal regressions were applied, controlling for measurement period, Cohort, and student enrollment size.

**Results:**

Strategy intensity scores ranged from 0.3 to 3.0 with 20% considered “higher-scoring” (score > 2.1) and 47% considered “lower-scoring” (< 1.2). Average MCI intensity scores more than tripled over the evaluation period, rising from 14.8 in the first grant year to 32.1 in year 2, 41.1 in year 3, and 48.1 in year 4. For each additional point increase in average MCI intensity score, the number of days per week that students reported PA for at least 60 min increased by 0.010 days (*p* < 0.01), and the number of hours per weekday that students reported engaging in screen time strategies decreased by 0.006 h (*p* < 0.05). An increase of 50 points in MCI intensity score was associated with an average 0.5 day increase in number of weekdays physically active and an increase of 55 points was associated with an average decrease of 20 min of sedentary time per weekday.

**Conclusions:**

We found a correlation between intensity and PA and sedentary time; increased PA and reduced sedentary time was found with higher-intensity MCIs. While additional research is warranted, practitioners implementing MCIs, especially with limited resources (and access to population-level behavior data), may consider intensity scoring as a realistic and cost effective way to assess their initiatives. At a minimum, the use of intensity scoring as an evaluation method can provide justification for, or against, the inclusion of an individual strategy into an MCI, as well as ways to increase the likelihood of the MCI impacting population-health outcomes.

**Supplementary Information:**

The online version contains supplementary material available at 10.1186/s12889-021-10312-y.

## Background

The health benefits of regular engagement in moderate-to-vigorous physical activity (PA) are well-established [1, 2]. Meeting the recommended 60 min of PA per day has musculoskeletal and cardiovascular health benefits and supports the maintenance of healthy body weight among children [3, 4]. More specifically, regular PA decreases the likelihood of becoming overweight or obese [1], with some estimates suggesting by as much as 70% [5]. In contrast, Tremblay et al. [5] found sedentary behavior significantly increases the likelihood of becoming overweight or obese by as much as 61%. Furthermore, evidence has emerged that sedentary behaviors are ubiquitous and represent a health risk independent of PA [6].

Traditional public health efforts have primarily targeted the individual, emphasizing education and behavioral skills training, and have not resulted in sustained behavior change [7, 8]. Consequently, there is an increasing focus on multi-component initiatives (MCIs), or efforts comprised of numerous strategies that work on multiple levels (i.e., individual, interpersonal, organizational, and community) at the same time, and in a best case scenario are synchronized across levels [9]. Such initiatives tend to target multiple settings such as schools, parks, and streets simultaneously—and do so in a coordinated manner to create greater intensity, effect, and sustainable change [9–19].

To ensure success, MCIs must include evidence-based strategies, as well as extensive community engagement and attention to community needs, wants, and strengths [20]. This kind of organization and coordination of strategies across different settings is challenging, yet there are a number of successful MCIs [10–13, 15–17, 21]. For example, Romon et al., [13] found that over a long period of time, MCIs with various strategies (e.g., new sporting facilities built, educators employed to promote PA in schools, and family strategies organized) had a synergistic effect on overweight prevalence. Shape Up Somerville — a comprehensive intervention that included a variety of school and community-wide initiatives — was found to decrease BMI z-score in children at high risk for obesity [22].

Assessment of MCIs prove to be very challenging [18, 23], and rigorous evaluations (e.g., randomized control trial, quasi-experiment) may not be viable especially when assessing the long-term effectiveness of MCIs. Moreover, while funders and other stakeholders want to see population-level health improvements (e.g., reduced obesity), these changes may not be detectable in the short-term [24, 25]. Efforts to assess strategies and their likely positive impact in the long-term are warranted, and have been increasingly explored [15, 26–30]. Cheadle et al. [31], developed and assessed various attributes (e.g., reach, efficacy and strength) of single strategies, and found those with higher reach and strength were correlated with improved health behaviors [31].

Numerous other researchers collaborated to explore the relationship between child obesity and characteristics of 130 MCIs across the U.S. using three predictors of population health—the purpose (the way the strategy is intended to impact behavior), duration (the length of time of the strategy), and reach (the number of people exposed to a strategy) [15, 27]. Each strategy was assessed for purpose, duration, and reach, and an overall MCI “intensity” score was calculated, based on the science which suggests that lower scores have less likelihood for sustained population-level behavior change compared to higher scoring strategies. Strategies that improve access (e.g., installation of walking/running trail), reduces barriers (e.g., increased time in physical education classes), or changes broader conditions (e.g., lighting repairs and maintenance of neighborhood playground) are higher in intensity compared to those aiming to educate or enhance skills (e.g., information on how to be physically active) [32–34]. Evidence also suggests that when more people are exposed, and for longer periods of time, the greater the likelihood that the strategy will lead to desired outcomes [27, 28, 31].

These types of studies have made an impactful contribution to the field by establishing a systematic way for measuring MCIs. That said, further exploration of these methodologies as a tool for assessing MCIs, determining the intensity necessary to achieve health improvements, and making strategic improvements is important.

### Purpose and objectives

Using a realistic and cost effective way to assess MCIs, the purpose of this study was to explore intensity scoring as described by Fawcett et al. [28] and Collie-Akers et al. [27] to assess whether higher intensity PA-related MCIs implemented as part of the Healthy Schools Healthy Communities (HSHC) initiative were associated with improved physical activity-related behaviors among youth.

## Methods

### Intervention approach

With funding from the Missouri Foundation for Health, HSHC was implemented across 34 Missouri school districts and their respective communities to address childhood obesity targeting thousands of children and youth in grades K–8. Funding was intended to build on existing school and community assets to stimulate implementation of new and/or advanced efforts for increasing access to healthy food and PA in vulnerable communities throughout the foundation’s catchment area (see https://mffh.org/the-foundation/where-we-work/). As per the logic model, technical assistance and increased linkages within and across grantees, resources, and funding were intended to lead to short-term outcomes (e.g., establishment of strong, durable partnerships; regular collaboration and communication), intermediate outcomes (e.g., increased capacity, improved perceptions and behaviors regarding PA), and ultimately, long-term outcomes (e.g., increased percentage of youth at a healthy weight). HSHC began in 2013 with a cohort of 13 school districts across 12 communities (Cohort 1). The Missouri Foundation for Health enrolled 12 new school districts (adding one new community) (Cohort 2) in 2014, and 9 new school districts (Cohort 3) in 2015. Overall, there were 33 school districts enrolled across 13 communities included in the analysis.

School and community coordinators conducted wellness assessments and created action plans to achieve the long-term goal of reducing childhood obesity. School action plans were guided by the Alliance for a Healthier Generation’s Healthy Schools Program Framework of Best Practices (Alliance Framework) [35] and addressed CSPAP components. Community action plans were informed by the YMCA’s Community Healthy Living Index (CHLI) [36]. Across schools and communities, both the action plans and stakeholders implementing the strategies varied greatly, however they all included any combination of CSPAP strategies, and aimed to: 1) increase knowledge and awareness, enhance skills, support behavior change, and motivate the community, and 2) modify broader conditions. Common strategies were walk-to-school days, health and wellness fairs, joint-use-agreements, and installation of playground equipment or walking trails. Table 1 provides examples and an overview of these strategies, as recorded by school and community coordinators in real-time, by year.

**Table 1:** Activity Types Implemented Over the First Four Years, Missouri HSHC^a^ 2013–2017

Type	Purpose	Examples^a^	Measure	Strategies and Population Reach^c^				
				Year 1 2013–2014	Year 2 2014–2015	Year 3 2015–2016	Year 4 2016–2017	**Total**
Programs & events^b^	Strategies to provide information, enhance skills, & support behavior, practice, policy, and environmental change	• Walking/running clubs • Walk-to-school days • Health and wellness fairs	programs/events (n)	145	388	560	610	1703
			people reached^c^ (n)	36,910	118,337	118,748	138,217	412,212
Practice, policy, and environment change^b^	Changes or improvements in operations, documents, or infrastructure to increase access, improve places, and ensure sustainability	• Teachers implement classroom physical activity breaks • Trails used to provide hands-on learning • Joint-use agreements signed • School wellness policies & city/town plans revised • Trails/tracks/sidewalks built • Playground equipment installed	strategies (n) people reached^c^ (n)	18 1567	123 58,585	209 310,565	121 431,113	471 801,830

^a^Across each multi-component initiative any combination of these strategy types were implemented at varying times throughout the four years

^b^ Programs/events and practice, policy, and environmental changes presented here only include those with a focus on physical activity and/or physical activity and healthy eating

^c^Reach was defined as the number of individuals who either participated (e.g., program) or could have been exposed based on where the intervention was implemented (e.g., school wellness policy)

John Snow, Inc. (JSI), a research and consulting firm specializing in the implementation and evaluation of community-wide initiatives, was contracted by the Missouri Foundation for Health to conduct a mixed-methods evaluation during the first half of the HSHC initiative (2013–2017). This study was reviewed by John Snow Inc. IRB (OHRP IRB00009069) and deemed exempt. The evaluation was guided by the work of HSHC logic model and assessed the strategies set forth in each district and respective community’s action plans. Various methods were used to capture both quantitative and qualitative data including: 1) an online data platform which allowed grantees to document their strategies in real-time; 2) interviews with grantees; and 3) surveys administered at school to all students in 5th through 8th grades at baseline (in the fall) and once a year thereafter (in the spring) to assess PA behaviors and perceptions.

**Fig. 1:**
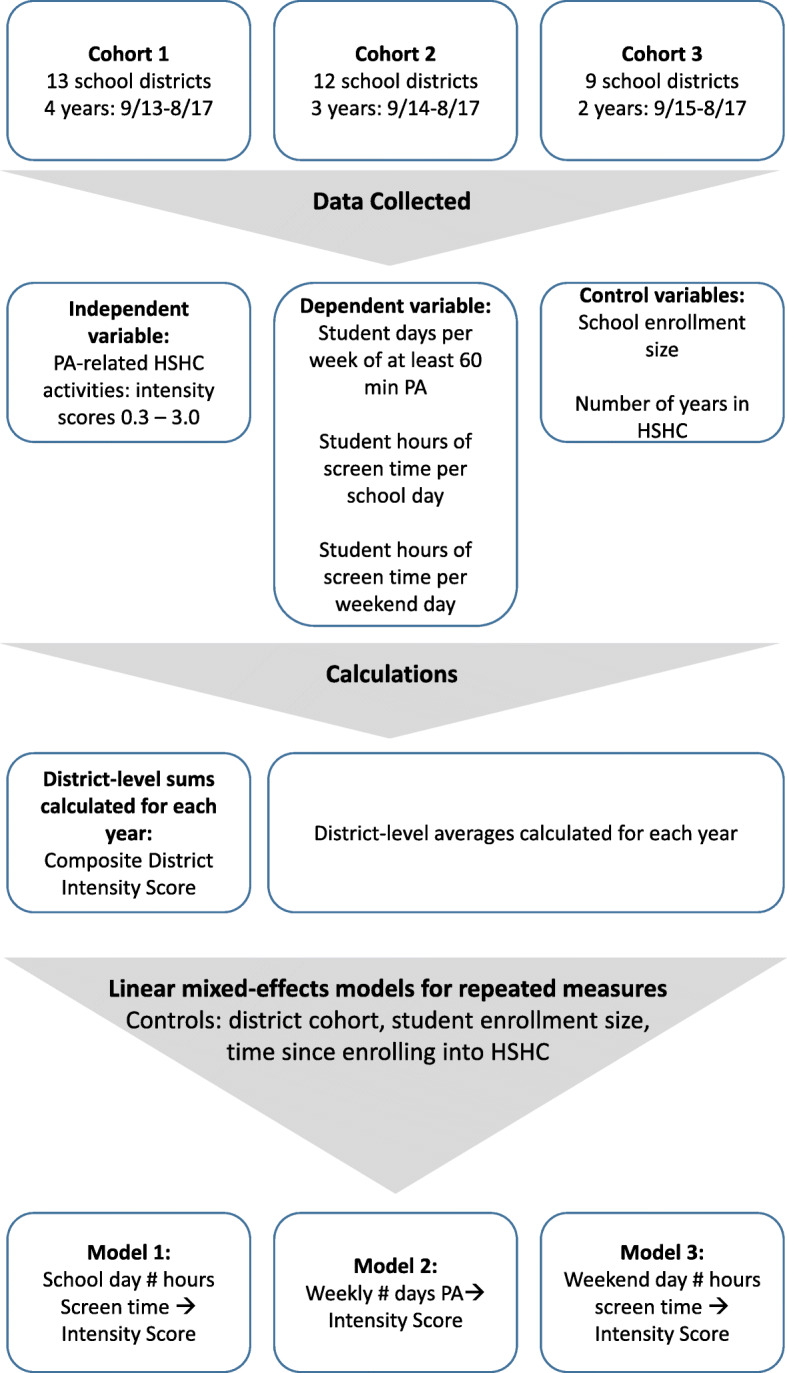
Evaluation Overview

Regardless of how intentional or coordinated, each strategy was included in the evaluation if it was reported by the coordinators. For this investigation, however, strategies were only included in the analysis if they specifically targeted PA and/or sedentary activity across the participating schools and communities (Table 1). Strategies that only addressed healthy eating and those that were not reported by local champions between September 1, 2013 and July 31, 2017, were not included in these analyses (see Fig. 1 for an evaluation overview).

### Independent variables

#### Strategy intensity score

Individually, four evaluation team members assigned point values for every strategy based on the three attributes used in previous research [27]: 1) purpose (i.e., providing information, enhancing skills or services, modifying access or changing broader conditions); 2) duration (i.e., occurring just once, several times, or ongoing); and 3) reach or penetration of the strategy (i.e., the proportion of the total city/town population that either participated in or could have been exposed to the strategy based on where the intervention was implemented). To ensure inter-rater reliability, an agreement across the study team of at least 80% was accomplished. Each attribute was scored either a 0.1 (minimum), 0.55 (medium), or 1.0 (maximum) and summed to calculate an intensity score for every strategy (Σ purpose value + duration value + reach value). Strategy scores ranged from 0.3 (weakest and potentially of less influence on longer-term outcomes) to 3.0 (strongest and potentially more sustainable and of greater influence). Strategies that were a more permanent fixture (e.g., new playground equipment) or a policy that would take a process to change (e.g., a joint use agreement) were scored “ongoing” (assigned a duration score of 1.0). Strategies defined as ongoing were treated as “active” in each subsequent year after its reported adoption/installment, unless otherwise reported as over. For example, if a new park was installed in 2014, it would be assigned a duration score of 1.0 and its intensity score would be included as a separate strategy in the 2015, 2016, and 2017 grant years. Scoring examples are provided in Table 2.

**Table 2:** Protocol for Assigning Intensity Score for Each Strategy, Missouri HSHC 2013–2017

Dimension	Rubric for Scoring Intensity (0.1 = low;1.0 = high)	Related Examples
**Purpose**	**High (1.0):** Modifying policies, systems, and access **Med (0.55):** Enhancing services and support **Low (0.1):** Providing information; enhancing skills	**High**: A long-term transportation plan was passed and included a sales tax to generate revenue to support pedestrian and transportation infrastructure **Med**: PA opportunities were offered as a reward in place of food but was not a mandated policy **Low**: Fun fit night for families to engage in activities together
**Duration**	**High (1.0):** Ongoing, throughout the year **Med (0.55):** More than once per year **Low (0.1):** One-time event	**High**: Joint-use agreement in place to enable school grounds to be used for PA after school hours **Med**: Walking challenge occurring twice throughout the year **Low**: 5 k Run
**Reach**^a^	**High (1.0):** 21% or more of the population **Medium (0.55):** 6–20% of the population **Low (0.1):** 0–5% of the population	**High**: Livable Streets policy **Medium**: Revised wellness policy impacted schools districtwide **Low**: A rock wall was installed at a middle school

^a^Targeted population was calculated using U.S. Census data for each targeted city/town

#### MCI intensity score

An average composite intensity score comprised of all strategies reported for each school district and its respective community was calculated and reported as the MCI intensity score. In cases where multiple school districts were located in the same community, or served by the same community-based organization funded to implement HSHC strategies, the individual community-level intensity scores were included in each school district’s MCI intensity score. Separate MCI intensity scores were calculated for each district for every grant year of participation.

#### District cohort

HSHC began with a cohort of 13 school districts across 12 communities in fall 2013 (Cohort 1) and was expanded by the Missouri Foundation for Health over the next 2 years. In 2014, HSHC grew to include 12 new school districts (adding one new community) (Cohort 2); in 2015, an additional 9 school districts were added (Cohort 3).

#### Time since enrolling in HSHC (Grant year)

Grant year accounts for the number of years that the cohort or group of school districts was enrolled in HSHC. Because school districts were enrolled in HSHC between the of fall of 2013 and 2015, the baseline of zero grant years (and subsequent years) corresponds to different calendar years depending on each school district’s cohort. The first grant year for Cohort 1 was 2013, Cohort 2 was 2014, and Cohort 3 was 2015. For example, the first grant year for Cohort 1 took place in 2013, and thus the time since enrolling in HSHC would be equal to 0. In 2014, it would be equal to 1, while Cohort 2 (which started in 2014) would be equal to 0.

#### Student enrollment size

The total number of students enrolled in the targeted grades for HSHC (K–8) was determined annually by the Missouri Department of Elementary and Secondary Education’s website (https://mcds.dese.mo.gov/quickfacts/Missouri School Directory). School districts were classified into a continuous format ranging from 1 to 63 for every 100 students (enrollment sizes of 1–100 students were assigned a value of 1, sizes of 101–200 students a value of 2, etc.).

### Dependent variables

*PA.* A self-reported survey was administered by classroom teachers to all 5th through 8th grade students enrolled in the school districts. The survey was conducted in the spring of year 1 enrollment into HSHC (which was different for each cohort) and each subsequent school year. Standard questions on PA behaviors and perceptions were incorporated into the survey. Prior to administering the survey, the reading level of each survey question was verified (which averaged a 6th grade reading level) and piloted with a number of 5th and 6th graders. Students were asked, “*During the past 7 days, on how many days were you physically active for a total of at least 60 minutes per day? Add up all of the time you spent in any kind of physical activity that increased your heart rate and made you breathe hard for some of the time.”* PA time was defined as the number of days students reported engaging in PA for at least 60 min.

#### Screen time

Using the same self-reported survey as defined above, students were asked about their screen time (a major form of sedentary behavior) on an average school and weekend day. The questions were informed by the literature documenting recommended assessments of sedentary behaviors [37]. Questions read the same with the exception of the day (school day and weekend day). *On an average* *“X” day, how many hours do you watch TV, play video games or computer games, or use a computer for something that is not school work? Count the time spent on things such as Xbox, PlayStation, an iPod, and iPad or other tablet, a smartphone, YouTube, Facebook or other social networking tools like Twitter or Pinterest, and the internet).”* Screen time was defined as the number of hours per school and weekend day spent engaging with such technology.

Outcomes were operationalized as the average district number of days reported being physically active for a total of at least 60 min per day during the past 7 days, and average district number of hours of screen time per school and weekend day.

### Analysis

Data were aggregated at the school-district level for each school year, beginning September 1, 2013 through July 31, 2017. This includes both school and community strategies. Descriptive statistics were generated to describe the MCI intensity scores and each of the three outcomes over time and by cohort. ANOVAs were applied to detect significant differences between cohorts and outcome measures at baseline. Pearson’s correlation was conducted to evaluate the crude association between MCI intensity scores and the three outcomes. To account for the correlation of repeated measurements within school districts, number of days per week physically active, and both number of hours engaged in screen time outcomes were analyzed using linear mixed-effects models for repeated measures (repeated observations nested within districts). Models included a random effect for district. All models controlled for district cohort (1, 2, or 3), student enrollment size, and time since enrolling into HSHC. Controlling for these factors is important given the variations among schools and communities and the time within which they entered into HSHC. A variance components covariance structure was used under the assumption of independence across measures and due to the fact that the independent variables were measured along different scales. With time since enrolling into HSHC as a fixed effect, the Bonferroni correction was applied to adjust for multiple comparisons over time. Analyses were conducted in SAS 9.4 (SAS Institute, Inc. Cary, NC).

## Results

### PA-related strategies by CSPAP

**Fig. 2:**
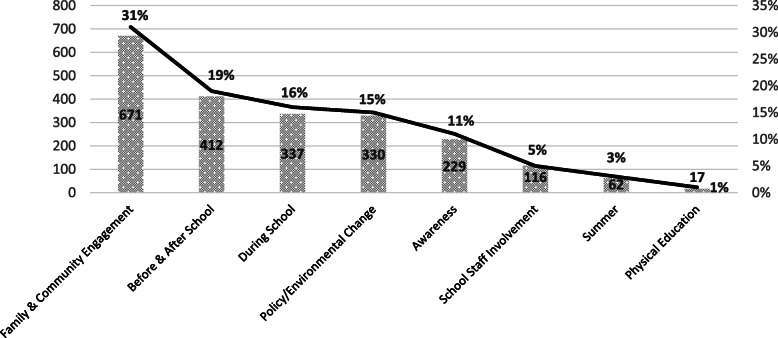
Total Number and Percentage of Strategies by Comprehensive School Physical Activity Program (CSPAP) Interventions^a^. ^a^ The CSPAP framework organizes school physical activity interventions into five categories: 1) physical activity before and after school, 2) physical activity during school. 3) family and community engagement, 4) physical education, and 5) school staff involvement

There were 2174 PA-related strategies implemented over the 4 years of study (Fig. 2) – see the supplementary table for breakdown of the PA-related strategies by community and school/childcare across time. Almost one-third (31%) of the strategies were community-based; 19% of the strategies were related to before and after school physical activity, with the majority occurring primarily after school; and 16% were related to increased physical activity during school. Fifteen percent (15%) of the strategies were either environmental or policy changes which occurred in the school and/or community.

### PA-related score by attribute

Out of the 2174 PA-related strategies implemented from 2013 to 2017, 56.2% were low-scoring in purpose (0.1), 31.6% were medium scoring (0.55), and 12.2% were high-scoring (1.0). In other words, over half of the strategies aimed to increase knowledge or enhance skills, one-third enhanced services, and a little over 10% modified access or changed broader conditions. Almost half (49.2%) of all strategies were low-scoring in duration, meaning they happened only once. Just over one-third (39.1%) were medium-scoring, and happened more than once but were not ongoing, and 11.7% were high-scoring or more permanent strategies. Finally, among the reach attribute score components, 51.7% were low-scoring, meaning they reached less than 5% of the total population, and almost one-quarter (24.4 and 24.0%, respectively) were both medium and high-scoring, and reached higher percentages of the population.

### PA-related score by strategy

The sum of each individual strategy’s attributes, or PA-related strategy score ranged from 0.3 (lowest) to 3.0 (highest), with 20% of strategies considered “higher-scoring” (2.1 or higher), and an increased likelihood of having a greater impact on long-term positive behavior change and health outcomes. Almost half of all strategies (47%) were “lower-scoring” (score of 1.2 or below). Across all years and districts, the average individual strategy score was 1.33.

### MCI intensity scores

The MCI intensity scores, or the averaged sum of all the strategies implemented within a school district and its respective community rose from 14.8 in the first grant year to 32.1 in year 2, 41.1 in year 3, and 48.1 in year 4 (Table 3). The total mean MCI intensity score for all Cohort 1 districts increased with every subsequent year engaged in HSHC (with the exception of the last year where it remained steady from the previous year), rising from a mean of 15.4 in the first year to 48.1 in the fourth year. Cohort 1 was involved in HSHC for the longest time, and scores were higher every grant year (2014–2017) compared to Cohorts 2 and 3. Where comparisons were possible, this trend held true for Cohort 2, which was enrolled in HSHC for the second longest period of time, and had higher scores than Cohort 3.

**Table 3:** Characteristics of Districts, Missouri HSHC 2013–2017

Year in HSHC (actual Year)	N	Number of days/week physically active for 60+ minutes (SD)	Number of screen time hours per weekday (SD)	Number of screen time hours per weekend day (SD)	Total Number of Strategies	Average MCI Intensity Score (SD)	Average Intensity Score per Strategy
**Baseline**		**4.4 (0.5)**	**2.4 (0.4)**	**3.1 (0.3)**	**0.0**	**0.0**	**0.0**
Cohort 1 (2013)	13	4.2 (0.4)	2.5 (0.3)	3.2 (0.2)	0.0	0.0	0.0
Cohort 2 (2014)	12	4.7 (0.5)	2.3 (0.4)	3.1 (0.3)	0.0	0.0	0.0
Cohort 3 (2015)	9	4.2 (0.5)	2.4 (0.5)	3.0 (0.4)	0.0	0.0	0.0
**Year 1**		**4.8 (0.5)**	**2.4 (0.4)**	**3.0 (0.3)**	**417**	**14.8 (8.7)**	
Cohort 1 (2014)	13	4.7 (0.4)	2.5 (0.3)	3.1 (0.2)	163	15.4 (10.3)	1.24
Cohort 2 (2015)	12	5.1 (0.6)	2.4 (0.3)	3.0 (0.3)	144	15.3 (8.3)	1.27
Cohort 3 (2016)	9	4.7 (0.6)	2.4 (0.5)	2.9 (0.3)	110	13.4 (7.2)	1.04
**Year 2**		**4.7 (0.6)**	**2.5 (0.3)**	**3.0 (0.3)**	**836**	**32.1 (15.5)**	
Cohort 1 (2015)	13	4.7 (0.3)	2.5 (0.3)	3.1 (0.3)	377	37.0 (19.8)	1.28
Cohort 2 (2016)	12	5.0 (0.9)	2.6 (0.4)	3.0 (0.3)	248	30.6 (12.4)	1.48
Cohort 3 (2017)	9	4.3 (0.5)	2.5 (0.4)	3.0 (0.3)	211	27.0 (11.1)	1.12
**Year 3**		**4.9 (0.5)**	**2.5 (0.4)**	**3.0 (0.3)**	**730**	**41.1 (19.1)**	
Cohort 1 (2016)	13	4.8 (0.3)	2.6 (0.4)	3.1 (0.4)	478	50.2 (16.8)	1.37
Cohort 2 (2017)	12	5.1 (0.7)	2.4 (0.4)	3.0 (0.4)	252	31.2 (16.9)	1.48
**Year 4**		**4.8 (0.6)**	**2.6 (0.4)**	**3.1 (0.3)**	**454**	**48.1 (20.0)**	
Cohort 1 (2017)	13	4.8 (0.6)	2.6 (0.4)	3.1 (0.3)	454	48.1 (20.0)	1.38

**Fig. 3:**
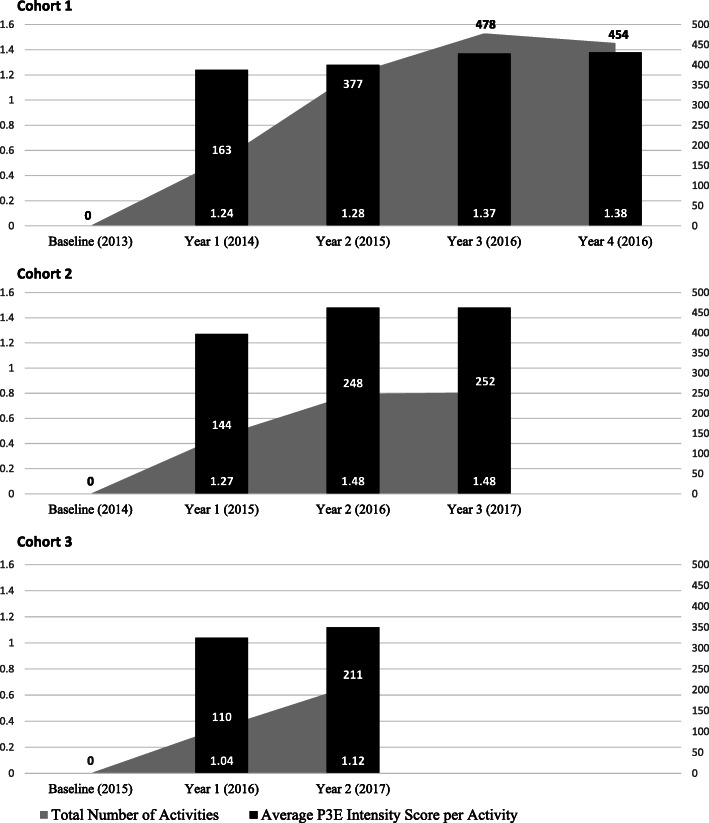
Total Number of Activities and Average Intensity Score per Activity

Across all years and cohorts, Cohort 1 had higher average MCI intensity scores, but implemented a considerably higher number of strategies compared to Cohorts 2 and 3 (Fig. 3). Moreover, while the actual number of Cohort 1 strategies more than doubled in the second year compared to the first (*n* = 377 vs. 163), the average strategy intensity score only increased slightly (1.28 from 1.24). In the third year, Cohort 1 implemented 478 strategies compared to Cohort 2’s 252, but the average intensity score per strategy was lower (1.37 vs. 1.48, respectively). Cohort 1 was the only one involved all 4 years of the study, with 454 strategies implemented in year 4, averaging an intensity of 1.38 per strategy. In summary, the large number of Cohort 1 strategies implemented each year drove up Cohort 1’s average MCI intensity score.

Unlike Cohort 1, Cohort 2 implemented fewer strategies but those that were implemented were at a higher intensity per strategy. Cohort 2 strategies did not quite double in year 2 compared to year 1 (*n* = 248 vs. 144), but the average intensity score per strategy was higher in the second year compared to the first (1.48 from 1.27). The number of Cohort 3 strategies almost doubled in year 2 compared to year 1 (*n* = 211 vs. 110), but the strategies had the lowest average intensity score in both year 1 (1.04) and year 2 (1.12) compared to the other cohorts’ year 1 and 2 scores.

### PA and screen time behavior outcomes

It is likely that strategies were implemented prior to the launch of HSHC, however, they were not documented and therefore assumed as 0. The total mean number of days per week students reported PA for at least 60 min increased from 4.4 days at baseline to 4.8 days at year 4 (Table 3). While Cohort 1 showed a 0.6 day increase from baseline to year 4, Cohorts 2 and 3 showed a 0.1 day increase from baseline to years 3 and 2, respectively. The mean number of screen time hours per weekday and weekend day remained relatively stable across all study years for all cohorts.

As indicated in Table 3, there were no significant differences at baseline across cohorts in mean number of days per week that students reported PA for at least 60 min per day (*p* = 0.1004), or number of screen time hours reported per school (*p* = 0.4743) or weekend day (*p* = 0.2711).

At baseline, the average number of days students reported engaging in PA 60 min or more per day over the past 7 days was 4.4. Cohorts 1 and 3 reported fewer days compared to Cohort 2 (4.2 days vs. 4.7, respectively). The number increased for each cohort after its first year in HSHC (average 4.8 days compared to 4.4 days at baseline) and remained higher than baseline each year thereafter. The number of hours engaged in screen time on the average weekday increased slightly from baseline, with reported cohort averages ranging from 2.3 to 2.6 across all cohorts and survey years. Students reported engaging in more hours of screen time on a weekend day, compared to a weekday, with averages ranging from 2.9 to 3.1.

### Relationship between MCI intensity scores and PA and screen time

Multivariate longitudinal regressions were applied to better understand the association between average MCI intensity score and PA and screen time, controlling for measurement period, cohort, and student enrollment size (Table 4). There was a statistically significant positive correlation between the average MCI intensity score and the number of days per week that students reported PA for at least 60 min per day. For each additional point increase in average MCI intensity score, the number of days per week students reported PA for at least 60 min increased by 0.010 days (*p* = 0.004). In other words, holding cohort, student enrollment size, and grant time constant, a modeled increase of 50 points in the average MCI intensity score (achievable through implementation of multiple MCI strategies within the same district) is associated with an average increase of 0.5 days per week of PA for at least 60 min.

**Table 4:** Summary of multiple longitudinal regression analysis, Missouri HSHC 2013–2017

Model 1: Number of days per week physically active 60 + minutes				
	β	SE	99% CI	*p*-value^a^
Constant	4.437	0.11	4.295 to 4.670	< 0.0001
**Average MCI Intensity Score**	**0.010**	**0.00**	**0.003 to 0.017**	**0.004**
Baseline (*ref*)				
1 year	0.257	0.13	−0.009 to 0.522	0.058
2 years	−0.040	0.16	−0.367 to 0.286	ns
3 years	0.039	0.20	−0.355 to 0.432	ns
4 years	0.035	0.23	−0.421 to 0.491	ns
Cohort 1 (*ref*).				
Cohort 2	0.342	0.11	0.125 to 0.559	0.003
Cohort 3	0.050	0.13	−0.214 to 0.314	ns
Enrollment size	−0.015	0.00	−0.025 to − 0.006	0.002
**Model 2: Number of screen time hours per weekday**				
	**β**	**SE**	**99% CI**	***p*-value**^a^
Constant	2.420	0.08	2.256 to 2.583	< 0.0001
**Average MCI Intensity Score**	**−0.006**	**0.00**	**−0.011 to − 0.001**	**0.016**
Baseline (*ref*).				
1 year	0.123	0.09	−0.065 to 0.310	ns
2 years	0.308	0.12	0.078 to 0.539	0.009
3 years	0.339	0.14	0.061 to 0.618	0.018
4 years	0.394	0.16	0.071 to 0.716	0.017
Cohort 1 (*ref*).				
Cohort 2	−0.108	0.07	−0.261 to 0.044	ns
Cohort 3	−0.135	0.09	−0.321 to 0.051	ns
Enrollment size	0.006	0.00	−0.001 to 0.012	0.080
**Model 3: Number of screen time hours per weekend day**				
	**β**	**SE**	**99% CI**	***p*-value**^a^
Constant	3.131	0.07	2.993 to 3.269	< 0.0001
**Average MCI Intensity Score**	**−0.003**	**0.00**	**−0.007 to 0.001**	**0.098**
Baseline (*ref*).				
1 year	−0.060	0.08	−0.218 to 0.098	ns
2 years	0.033	0.10	−0.161 to 0.227	ns
3 years	0.049	0.12	−0.186 to 0.283	ns
4 years	0.113	0.14	−0.159 to 0.385	ns
Cohort 1 (*ref*)				
Cohort 2	−0.079	0.06	−0.208 to 0.050	ns
Cohort 3	0.173	0.08	−0.330 to − 0.017	0.031
Enrollment size	0.007	0.00	0.001 to 0.012	0.022

^a^*p*-values for the differences in grant time (fixed effects) least squares means were adjusted for multiple comparisons using the Bonferroni correction. All analyses conducted in SAS 9.4 (SAS Institute, Inc. Cary, NC)

There was also a statistically significant negative correlation between the average MCI intensity score and the number of hours per weekday students reported being engaged in screen time. For each additional point increase in average MCI intensity score, the number of hours per weekday that students reported engaged in screen time strategies decreased by 0.006 h (*p* = 0.016). In other words, holding cohort, student enrollment size, and grant time constant, a modeled increase of 55 points in the average MCI intensity score (achievable through implementation of multiple MCI strategies within the same district) is associated with an average decrease of 20 min of screen time per weekday. Even though average number of weekday hours spent engaged in screen strategies increased slightly over time, the relationship between higher intensity scores and lower screen time was significant. There was also a negative correlation between weekend screen time hours per day and intensity score, but it was not significant (*p* = 0.098).

## Discussion

The comprehensive nature of MCIs, with multi-purpose PA strategies, show promise in addressing childhood obesity. Yet, they are extremely challenging to evaluate. Given the timing of the initiative (e.g., first 4 years) and the challenges of detecting changes in long-term outcomes such as obesity, the purpose of this study was to use intensity scoring to assess whether higher intensity MCIs implemented as part of a statewide initiative were associated with improved PA and reduced screen time among youth (dependent variables). Similar to previous research,^25, 26, 29^ we found a statistically significant relationship between higher MCI intensity scores and increased PA and decreased screen time behavior. Specifically, children living in a community with a higher average MCI intensity score had increased PA and decreased screen time. These findings are consistent with Pate et al. [38] who discovered a higher intensity scoring index for PA strategies was positively associated with non-Hispanic white children’s PA.

From a practical standpoint, an increase of 50 points was associated with an average 0.5 day increase in number of days per week physically active and an increase of 55 points was associated with an average decrease of 20 min of screen time per weekday. Yet, strategies contribute differently (between 0.3 to 3.0 points) to the overall MCI intensity score, and efforts to reach 50 to 55 points and see these results can also vary. The average contribution of each intervention across all districts and years was medium score of 1.33 points (constructed on a range with the lowest score possible being a 0.3 and the highest score possible a 3.0). Based on our findings, MCIs would need to include 38 medium-scoring strategies to decrease screen time by 20 min per weekday and/or include 42 medium-scoring interventions to increase 60 min of daily physical activity by 0.5 days per week. Higher MCI intensity scores (> 50 points) can be accomplished by implementing 1) strategies that modify access or change broader conditions which reach more people over longer periods of time (e.g., policy/environmental changes), 2) a greater number of strategies that increase knowledge or enhance skills but reach fewer people for shorter periods of time, or 3) a combination of both. Given that resources are often limited, careful consideration of the strategies within any given MCI should be taken to maximize the likelihood of long-term impact on population-level health.

In a review of three MCI case studies, Mikkelsen et al. [39], found that using the full range of strategies are key to a successful implementation, and efforts to increase knowledge, enhance services, modify access, and change broader conditions should all be included. The HSHC MCIs included a variety of educational events/programs, as well as policy, practice, and environmental changes, but there were over three times more programs and events as compared to policy, practice, and environmental changes (1703 vs. 471, respectively). Although programs and events are important, over half of the HSHC strategies scored low in purpose. Based on our findings, a more even distribution of strategies, fewer lower scoring strategies and more medium-to-high scoring strategies would have led to higher overall MCI intensity scores and likely better improvements in physical activity and screen time outcomes. Furthermore, while the goal of HSHC was to reduce childhood obesity in targeted communities, and the action plans were informed by evidence-based guides, this study did not assess whether strategies within the MCI reinforced each other. Future evaluations of MCIs should not only look at the intensity of the strategies but also whether they are synchronized and reinforcing.

Practitioners implementing MCIs are all faced with the challenge of how to demonstrate impact on population-level health behaviors and outcomes while also being open to participatory approaches and co-creation with local stakeholders. Mikkelsen et al. [39], found in general that program evaluator’s focus on overall effects. Yet, unlike a controlled setting where all possible confounders can be eliminated, MCIs are unpredictable in timing and scope, and populations are exposed (or not) to a potentially causal factor or factors (e.g., a new trail, a policy to ensure children have recess). Moreover, rigorous evaluation designs are often not feasible for local practitioners to conduct. As such, research suggests the importance of gathering and reporting evaluative data through methods that are suitable and acceptable in terms of scientific standards and support a project with timely feedback [40] while also ensuring that they are low-cost and easy to administer [39]. We found that scoring evidence-informed attributes of all strategies within a MCI and calculating an intensity score addresses these factors. Assessing the intensity of strategies can demonstrate progress towards reaching long-term goals, which can take years to be realized, as well as provide timely feedback which can guide program improvements or redirect resources (e.g., to increase the intensity of a strategy or strategies).

Within HSHC, over half of the strategies aimed to increase knowledge or enhance skills, 49.2% happened only once, 51.7% reached less than 5% of the total population. Findings such as these can help stakeholders and funders improve strategies within a MCI (e.g., implement at a greater frequency, work with partners to increase the reach of a program), or provide reasoning as to why a strategy (low-intensity) may be consuming a lot of resources with little return (e.g., likelihood of long-term impact). Adjustments may free up resources to implement fewer, more intense efforts that are more likely to lead to improved outcomes. Additional evaluations should consider the use of intensity scoring for both assessing and redirecting action plans, as well as understanding the impact of a MCI on youth physical activity and sedentary behavior.

This study is not without limitations. First, data were analyzed at the school district level and limited in terms of the overall small sample of districts and number of districts per year. Different types of interventions have varying levels of impact, and thus using a MCI district-level intensity score of all strategies cannot differentiate between intervention types or combinations. Second, student behaviors were self-reported and reading comprehension levels vary between 5th and 8th graders. We took steps in ensuring the appropriateness of the survey: it was piloted with 5th and 6th graders and the survey questions were adapted from valid and reliable sources. Each question averaged a 6th grade reading level and it was deemed appropriate. While we could have used more age-appropriate versions for each grade, we felt it was more important to reduce the potential for error in terms of the coordinators and/or teachers administering the wrong survey to the wrong students (e.g., 8th grade version given to 5th graders). Additionally, using the same survey version made it more methodologically sound to allow for comparisons across grades over time. Third, many previous studies show differences in the level of physical activity and sedentary behavior by age and sex. We controlled for grade, which we felt was reasonable given that there were no significant outliers by age per grade but did not control for sex given the number of controls in our model. Future studies should perhaps consider this variable. Fourth, PA levels were based on a question asking about activity that “increased your heart rate and made you breathe hard for some of the time.” While participants might have underreported the time spent in moderate activity, we found no significant differences between self-reported PA and data collected via pedometers on 5th grade students (not reported here). Future research should consider including multiple sources of data.

Another limitation was that the data used to calculate intensity was self-reported by various school and community stakeholders. It may have been incomplete and/or subjective, and there were likely variances in reporting across individuals. The evaluation team did, however, take necessary steps to ensure consistent and quality data collection by providing guidance and training in various formats (e.g., webinars, protocols, one-on-one technical assistance) and reviewing the data regularly. Upon review, the evaluation team followed-up with HSHC coordinators for clarification as needed. It was, however, left to the HSHC coordinator to provide the final data. Sixth, HSHC’s primary goal was to reduce childhood obesity and focused on MCIs that included both physical activity and healthy eating strategies. The purpose of this investigation was to identify associations between intensity and PA/sedentary behavior – two more intermediate outcomes – rather than the longer-term outcome of obesity. Given the focus of PA/sedentary time, we only considered the strategies that targeted physical activity-related behaviors (even if they also included healthy eating). It is possible that the healthy eating-only strategies, which were being implemented at the same time, may have confounded the results. Finally, the evaluation team was not involved in the enrollment of school districts and communities, which further challenged an evaluation design with a comparison/control group. The number of districts expanded to a total of 33 by 2017, but covered most of the same communities as in the first grant year. Children attending the schools later enrolled into HSHC may have been exposed to community-level interventions implemented prior to their school being on-boarded to HSHC, and their behaviors may have been influenced and baseline data subsequently impacted. Moreover, the time in which any child could have been exposed to a strategy may have varied for similar reasons. It is difficult to avoid confounding factors in an evaluation such as this, where the evaluators have no control in enrollment and implementation, and where the funding is limited. Nevertheless, the study limitations listed above are not unique to MCI being implemented and evaluated in “real-time.”

Regardless of the limitations, this study adds to the growing literature on MCIs addressing physical activity and screen time behavior across multiple settings in various communities, and with stakeholders facilitating and co-constructing the strategies. Findings expand upon others’ efforts to identify a realistic and cost effective way to scientifically evaluate these complex MCIs. While additional research is warranted, practitioners implementing MCIs, especially with limited resources (and access to data on population-level behaviors), may consider intensity scoring as a realistic and cost effective way to assess their initiatives. At a minimum, the use of intensity scoring as an evaluation method can provide justification for, or against, the inclusion of an individual strategy into an MCI, as well as ways to increase the likelihood of the MCI impacting population-health outcomes.

## Conclusions

MCIs are difficult to evaluate given the variations within which they are implemented. Findings from this study suggest the value of a systematic scoring approach in assessing MCIs aimed to address physical activity and screen time behavior. In addition to providing a scientific way to evaluate complex initiatives, intensity scores can provide justification for, or against, an individual strategy and ways to increase the likelihood of the MCI impacting population-health outcomes.

## Supplementary Information

**Additional file 1: Table S1.** 2174 MCI community (*N* = 982) and school/childcare (*N* = 1192) related strategies over time by cohort/school district.

## Data Availability

The authors do not wish to make the dataset available for several reasons: 1) it is the intellectual property of the authors and 2) the data were collected at the school level. Some of the schools were in rural areas and it is important that the schools (and students) remain anonymous.

## Abbreviations

B/A PA: PA before and after school
CDC: Centers for Disease Control and Prevention
CHLI: Community Healthy Living Index
CSPAP: Comprehensive School Physical Activity Program
HSHC: Healthy Schools and Healthy Communities
JSI: John Snow, Inc.
MCI: Multi-component initiative
PA: Moderate-to-vigorous physical activity
U.S.: United States
