# Supplementary material for: Association between multi-component initiatives and physical activity-related behaviors: interim findings from the Healthy Schools Healthy Communities initiative
Source: BMC Public Health. 2021 Feb 12;21:340. doi: 10.1186/s12889-021-10312-y (PMC7881474; doi:10.1186/s12889-021-10312-y)
Supplement: Supplementary file 1 — Additional file 1: Table S1. 2174 MCI community (N = 982) and school/childcare (N = 1192) related strategies over time by cohort/school district. [file 12889_2021_10312_MOESM1_ESM.docx]

| *Supplementary table: 2174 MCI community (N=982) and school/childcare (N=1192) related strategies over time by cohort/school district* | | | | | | | | |
| --- | --- | --- | --- | --- | --- | --- | --- | --- |
|  | **Year 1** | | **Year 2** | | **Year 3** | | **Year 4** | |
|  | **Community** | **School/Childcare** | **Community** | **School/Childcare** | **Community** | **School/Childcare** | **Community** | **School/Childcare** |
| **Cohort 1** | ***24 (0.96)*** | ***139 (1.28)*** | ***131 (0.97)*** | ***236 (1.38)*** | ***186 (1.10)*** | ***241 (1.27)*** | ***165 (0.75)*** | ***178 (1.12)*** |
| School #1.1 | 1 (0.75) | 12 (1.31) | 18 (1.00) | 26 (1.60) | 20 (1.02) | 29 (1.08) | 31 (0.56) | 30 (1.13) |
| School #1.2 | 4 (1.65) | 8 (1.82) | 14 (1.20) | 12 (1.65) | 14 (1.30) | 14 (1.33) | 29 (1.18) | 9 (1.10) |
| School #1.3 | 1 (1.65) | 4 (1.89) | 2 (0.98) | 10 (1.65) | 12 (1.01) | 22 (1.45) | 8 (0.75) | 12 (1.39) |
| School #1.4 | 5 (0.78) | 23 (1.20) | 16 (1.05) | 33 (1.30) | 20 (1.03) | 25 (1.00) | 5 (0.83) | 22 (1.02) |
| School #1.5 | 0 (0.00) | 15 (0.96) | 6 (0.90) | 27 (1.30) | 21 (0.81) | 26 (1.30) | 20 (0.53) | 14 (0.94) |
| School #1.6 | 7 (0.69) | 6 (2.25) | 9 (0.95) | 11 (1.81) | 2 (1.65) | 10 (2.10) | 15 (1.11) | 9 (1.70) |
| School #1.7 | 0 (0.00) | 4 (1.65) | 2 (1.20) | 12 (1.31) | 18 (1.63) | 4 (0.98) | 2 (0.98) | 9 (1.55) |
| School #1.8 | 0 (0.00) | 2 (1.43) | 4 (0.98) | 3 (1.80) | 7 (1.84) | 3 (2.25) | 1 (0.75) | 3 (0.90) |
| School #1.9 | 3 (1.20) | 34 (0.94) | 7 (0.69) | 38 (1.18) | 10 (0.84) | 43 (1.45) | 5 (0.48) | 17 (0.96) |
| School #1.10 | 0 (0.00) | 12 (1.16) | 10 (1.25) | 38 (1.38) | 6 (1.28) | 39 (0.89) | 8 (0.98) | 31 (0.85) |
| School #1.11 | 2 (0.30) | 0 (0.00) | 19 (0.49) | 8 (0.81) | 29 (0.91) | 7 (1.71) | 6 (0.45) | 1 (1.20) |
| School #1.12 | 1 (1.20) | 7 (1.07) | 7 (1.39) | 7 (0.88) | 7 (1.14) | 14 (1.23) | 4 (0.53) | 14 (1.23) |
| School #1.13 | 0 (0.00) | 12 (1.80) | 17 (1.01) | 11 (1.85) | 20 (1.02) | 5 (1.74) | 31 (0.56) | 7 (1.52) |
| **Cohort 2** |  |  | **70 (1.12)** | **74 (1.41)** | **124 (1.30)** | **108 (1.48)** | **120 (0.99)** | **68 (1.18)** |
| School #2.1 |  |  | 2 (1.20) | 2 (2.10) | 17 (1.65) | 4 (1.65) | 2 (0.98) | 1 (0.75) |
| School #2.2 |  |  | 2 (1.20) | 0 (0.00) | 17 (1.65) | 4 (2.21) | 2 (0.98) | 0 (0.00) |
| School #2.3 |  |  | 6 (1.58) | 6 (1.58) | 7 (1.14) | 14 (1.52) | 4 (0.53) | 1 (2.10) |
| School #2.4 |  |  | 11 (1.45) | 13 (1.06) | 14 (1.30) | 6 (1.13) | 29 (1.18) | 15 (1.23) |
| School #2.5 |  |  | 2 (1.20) | 3 (1.35) | 17 (1.65) | 3 (1.20) | 2 (0.98) | 1 (0.75) |
| School #2.6 |  |  | 6 (1.58) | 8 (1.31) | 7 (1.14) | 16 (1.51) | 4 (0.53) | 7 (1.52) |
| School #2.7 |  |  | 3 (1.50) | 16 (1.28) | 1 (3.0) | 10 (1.25) | 3 (0.75) | 0 (0.00) |
| School #2.8 |  |  | 17 (1.01) | 1 (1.20) | 20 (1.02) | 0 (0.00) | 31 (0.56) | 0 (0.00) |
| School #2.9 |  |  | 3 (1.05) | 5 (0.66) | 11 (0.55) | 8 (1.20) | 10 (1.56) | 21 (0.75) |
| School #2.10 |  |  | 8 (0.69) | 6 (1.35) | 0 (0.00) | 7 (2.23) | 14 (1.33) | 3 (1.05) |
| Westran #2.11 |  |  | 2 (0.30) | 8 (2.10) | 11 (0.91) | 31 (1.35) | 4 (0.86) | 13 (1.41) |
| Wheatland #2.12 |  |  | 8 (0.69) | 6 (2.10) | 2 (1.65) | 5 (1.83) | 15 (1.11) | 6 (1.73) |
| **Cohort 3** |  |  |  |  | **50 (0.81)** | **60 (1.23)** | **112 (1.02)** | **88 (1.07)** |
| School #3.1 |  |  |  |  | 7 (0.75) | 8 (1.71) | 21 (0.77) | 5 (2.10) |
| School #3.2 |  |  |  |  | 1 (0.30) | 12 (0.68) | 9 (0.55) | 18 (1.00) |
| School #3.3 |  |  |  |  | 0 (0.00) | 3 (2.10) | 12 (1.05) | 9 (0.90) |
| School #3.4 |  |  |  |  | 10 (0.62) | 5 (0.93) | 34 (1.31) | 0 (0.00) |
| School #3.5 |  |  |  |  | 10 (1.07) | 12 (1.28) | 7 (0.75) | 21 (1.22) |
| School #3.6 |  |  |  |  | 7 (1.14) | 0 (0.00) | 4 (0.53) | 1 (2.10) |
| School #3.7 |  |  |  |  | 1 (0.30) | 2 (1.20) | 5 (0.75) | 4 (0.98) |
| School #3.8 |  |  |  |  | 11 (0.55) | 7 (1.20) | 10 (1.56) | 21 (0.64) |
| School #3.9 |  |  |  |  | 3 (1.35) | 11 (1.36) | 10 (0.98) | 9 (1.35) |
| *Notes: Values reported are: number of strategies (average strategy intensity score) 252 unique strategies occurred across more than one school district, and are counted more than once in the above numbers and analysis. They account for 681 district strategies overall* | | | | | | | | |
